# Is health literacy of adolescent athletes’ parents whose children belonged to sports clubs related to their children’s intention to receive medications, vaccines, supplements, and energy drinks? A cross-sectional study

**DOI:** 10.1186/s12889-024-17746-0

**Published:** 2024-01-22

**Authors:** Rie Nakajima, Michihiro Komoriya, Fumiyuki Watanabe

**Affiliations:** Department of Pharmacy Practice in Primary Care, Nihon University School of Pharmacy, 7-7-1 Narashinodai, 274-8555 Funabashi-shi, Chiba Japan

**Keywords:** Health literacy, Adolescent, Athletes, Supplement, Medicine, Energy drink, Parents, Doping

## Abstract

**Background:**

Adolescent athletes’ values ​regarding health behaviors, including their attitudes toward doping, are largely derived from those of their parents. Therefore, clarifying the factors that affect parents’ intentions regarding their children’s medicine intake and nutrition can help elucidate the process of forming values ​​of healthy behaviors in young athletes.

**Methods:**

Between March 8 and March 9, 2021, an online questionnaire survey was conducted via an Internet research company; data from 2,000 residents in Japan were collected. Participants were male and female residents aged 30–59 years with children in elementary or high school and belonging to sports clubs. The survey items included respondent’s and child’s basic information, respondent’s health literacy, and level of sports in which the respondent and child were (or are) engaged. Respondents were also asked if they would like their children to receive prescription drugs, over-the-counter drugs, herbal medicines, vaccines, supplements, or energy drinks. Logistic regression analysis was performed to analyze the relationship between respondents’ basic information and health literacy and their intention to receive prescription and over-the-counter drugs, herbal medicines, vaccines, supplements, and energy drinks.

**Results:**

Higher parental health literacy was associated with higher children’s willingness to receive prescription drugs (odds ratio [OR] = 1.025, 95% confidence interval [CI]: 1.016–1.035), over-the-counter drugs (OR = 1.012, 95% CI: 1.003–1.021), prescription herbal medicines (OR = 1.021, 95% CI: 1.021–1.030), over-the-counter herbal medicines (OR = 1.012, 95% CI: 1.003–1.021), and vaccines (OR = 1.025, 95% CI: 1.016–1.035). Conversely, the children’s intention to receive energy drinks (OR = 0.990, 95% CI: 0.980–1.000) decreased significantly. As the child’s athletic level increased, parents’ willingness for their children to receive oral prescription medicines decreased (OR = 0.886, 95% CI: 0.791–0.992) and that to receive supplements (OR = 1.492, 95% CI: 1.330–1.673) and energy drinks significantly increased (OR = 1.480, 95% CI: 1.307–1.676).

**Conclusion:**

Health literacy of adolescent athletes’ parents is associated with their children’s willingness to receive medicines. Healthcare providers should counsel parents of adolescent athletes to allow their children to receive necessary drug treatments and prevent doping violations caused by supplement intake.

**Supplementary Information:**

The online version contains supplementary material available at 10.1186/s12889-024-17746-0.

## Background

In Japan, 65.2% of junior high-school students belong to sports clubs [1]. For these individuals, the time spent in sports clubs often occupies a large part of their daily lives. Motivations for adolescents joining sports clubs vary, with some seeking health benefits while others aspire professional athleticism. The participation of family members sports activities also plays a significant role in influencing children’s health attitudes and behaviors. For example, parents who are or have professionally engaged in sports may compel their children to participate at a level that is detrimental to their well-being [2]. Even in youth sports, when the level of competition increases, it is necessary to pay attention to athlete-specific health problems, such as participation in competitions subject to doping inspections. For example, past anti-doping rule violations have been reported not only for drugs but also for supplements; therefore, caution is required when consuming these drugs [3, 4]. Previous research suggests that adolescent athletes exhibit unique health behaviors. Some athletes refrain from excessive medicine intake or visiting hospitals because of the fear of violating the anti-doping rule, whereas others casually consume supplements and energy drinks [5–7].

Parents are responsible not only for their own lives and health but also for the health and welfare of their children. Children depend on their parents to prevent and manage their own health problems, and when parents lack knowledge and skills, their children’s health can be adversely affected [8, 9]. For example, previous research has shown that low parental health literacy has been associated with health problems in children, including obesity and poor oral health [10–12]. Thus, parents play an important role in maintaining their children’s health, and it is expected that their knowledge and awareness of their children’s health will have a significant impact on their children’s nutritional intake and how they deal with illness and injury. However, there is limited evidence that parental factors are involved in children’s willingness to take medicines and supplements.

For young athletes, the values that determine health behaviors, including doping behavior, are largely derived from those of their parents [13, 14]. Therefore, clarifying the factors that affect parents’ intentions regarding medicines, vaccines, supplements, and energy drink intake for their children would provide important information for elucidating the process by which adolescent athletes’ health values are formed. Therefore, in this study, we focused on factors that influence parental preferences regarding the intake of foods and medications related to the health of adolescent athletes.

We conducted a questionnaire survey for the parents of adolescent athletes who belonged to sports clubs and exercised daily and clarified the factors related to their willingness to receive medicines, vaccines, supplements, and nutritional drinks for their children.

## Methods

### Survey

A cross-sectional anonymous online questionnaire survey was conducted between March 8, 2021, and March 9, 2021. A questionnaire was distributed to males and females aged 30–59 years (approximately 198,218 people) residing in Japan who were registered with an Internet research company (Cross Marketing Co., Ltd.) and met the selection criteria (parents of elementary-school to high-school students who belonged to athletic clubs). The research firm in this study utilizes an unbiased panel of individuals representing various demographics, including age, sex, place of residence, marital status, occupation, and parental status [15]. Data from a total of 2,000 participants were collected by first-come, first-served in this study and evenly divided by sex and age. Response receipt was closed when the target number of respondents for each sex and age group was reached.

### Contents of the questionnaire

The survey items included basic information regarding respondent characteristics (sex, age, educational background, marital status, and number of children), level of sports activities (no competition, municipal-level, prefectural-level, and national-level competition), engagement to sports (previously or currently), and information regarding their children (sex, age, and level of sports). Respondents were asked to report on their most competitive child if they had multiple children.

The respondents’ intentions to receive prescription drugs, over-the-counter drugs, herbal medicines, vaccines, supplements, and nutritional drinks for their children were investigated using a 4-grade scale; (1) not wanting to receive at all, (2) not wanting to receive much, (3) want to receive, and (4) actively want to receive. Items related to the health literacy of the respondents were implemented in 16 items with reference to the Japanese version of the European Health Literacy Survey Questionnaire (HLS-EU)-Q47 [16, 17]. Each item was measured in five stages: 0. do not know, (1) very difficult, (2) somewhat difficult, (3) somewhat easy, and (4) very easy. The health literacy score was calculated using the following formula after treating “0. do not know/does not apply” on a 5-point scale as a missing value. To compare our findings with those of previous studies, we calculated the health literacy score based on the following formula, resulting in a final score ranging from 0 to 50 points. Health Literacy Score Criteria were divided into four levels: “poor” (0–25), “problematic” (> 25–33), “adequate” (> 33–42), and “excellent” (> 42–50).

Further, the Cronbach’s α coefficient was calculated to be 0.947, indicating the reliability of the self-report-based survey.

Health Literacy Score = (Average– 1) x (50/3) [17].

### Sample size

The G*Power software, version 3.1.9.7 (Heinrich-Heine-Universität, Düsseldorf, Germany) was utilized to determine the power of the sample size by logistic regression [18, 19]. Given the significance level set with Odds ratio = 1.3, Pr(Y = 1|X = 1) H0 = 0.2, α = 0.01, β = 0.95, R2 other X = 0 and X distribution = Normal, the required sample size should be at least 1,443 individuals.

### Analysis

A t-test and one-way analysis of variance were performed to determine the relationship between the respondents’ basic information and health literacy.

The respondents’ health literacy data were not normally distributed. Therefore, in this study, we used logistic regression, which is not related to normal distribution. Binary logistic regression analysis was performed to analyze the relationship between respondents’ basic information and health literacy and their intention to receive medicines, supplements, etc. for their children, and the odds ratio (OR) was calculated. The variables of the binomial logistic regression analysis were the respondents’ and children’s basic information and health literacy scores as independent variables, and the respondents’ willingness to receive medicines, vaccines, supplements, and energy drinks for children as dependent variables. Statistical software SPSS Statistics 27 (SPSS Inc., Chicago, IL, USA) was used for the analyses. Statistical significance was set at *P* < 0.05.

## Results

Responses were obtained from 2,000 participants. Table 1 shows the basic information such as sex, age, educational background, marital status, and athletic level of the respondents, as well as children’s sex, age, and athletic level. Overall, 48.5% respondents were male, and 51.5 were female with a mean age of 44.9 years (standard deviation [SD] ± 7.2). In addition, 62.9% of the respondents had graduated from a vocational school, university, or graduate school, and 63.4% of the children were male with a mean age of 12.7 years (SD 3.3). Among parents, no competition experience (38.2%) was most common, followed by municipal-level (30.9%), prefectural-level (23.3%), and national-level (7.7%) competition experiences. The children’s competition levels were in the order of municipal-level (43.5%), no (31.0%), prefectural-level (20.4%), and national-level (5.2%) competition experiences.

**Table 1:** Basic information about respondents and children

Characteristics		Total	%
		*n* = 2,000	
Parent’s sex	Male	970	48.5
	Female	1030	51.5
Parent’s age	30–39 years	624	31.2
	40–49 years	687	34.4
	50– 59 years	689	34.5
Parent’s educational status	Junior high school	42	2.1
	Senior high school	425	21.3
	Junior College/Technical College	276	13.8
	Vocational School/University/Graduate School	1257	62.9
Parent’s marital status	Unmarried	19	1.0
	Married	1981	99.1
Parent’s sports level	no competition experience	763	38.2
	experience in municipal-level competitions	617	30.9
	experience in prefectural-level competitions	466	23.3
	experience in national competitions	154	7.7
Number of children	1	941	47.1
	2	825	41.3
	3	205	10.3
	over 4	29	1.5
Children’s sex	Male	1267	63.4
	Female	733	36.7
Children’s sports level	no competition experience	619	31.0
	experience in municipal-level competitions	870	43.5
	experience in prefectural-level competitions	407	20.4
	experience in national competitions	104	5.2
Main sports engaged in by children	Soccer	440	22.0
	Baseball	222	11.1
	Tennis	190	9.5
	Basketball	189	9.5
	Volleyball	126	6.3
	Table Tennis	95	4.8
	Track and Field	94	4.7
	Swimming	93	4.7
	Others	583	29.1

Health literacy scores were calculated from the responses to the HLS-EU-Q16 questionnaire. Table 2 shows the average demographic characteristics and health literacy scores of the respondents. The average health literacy score was 25.4 (SD = 10.2). According to the health score classification, 981 (49.1%) respondents had poor health literacy, 557 (27.9%) had problematic health literacy, 346 (17.3%) had adequate health literacy, and 116 (5.8%) had excellent health literacy. The average health literacy score was 24.7 (SD 10.4) for males and 26.1 (SD 10.0) for females, which was significantly lower for males than for females (*p* = 0.003). Significant differences were also observed in the educational status (*p* = 0.028) and sports level (*p* = 0.002) of parents.

**Table 2:** Respondent demographics and health literacy score

Characteristics		Categories of Health Literacy of Parents				Mean ± SD	p-value
		Poor (0–25)	Problematic (> 25–33)	Adequate (> 33–42)	Excellent (> 42–50)		
		n(%)					
Total		981(49.1)	557(27.9)	346(17.3)	116(5.8)	25.4(10.2)	
Parent’s sex	Male	508(52.4)	240(24.7)	171(17.6)	51(5.3)	24.7(10.4)	0.003^a^
	Female	473(45.9)	317(30.8)	175(17.0)	65(6.3)	26.1(10.0)	
Parent’s age	30–39 years	303(48.6)	172(27.6)	108(17.3)	41(6.6)	25.7(10.6)	0.112^b^
	40–49 years	358(52.1)	185(26.9)	109(15.9)	35(5.1)	24.8(10.1)	
	50–59 years	320(46.4)	200(29.0)	129(18.7)	40(5.8)	25.8(9.9)	
Parent’s educational status	Junior high school	21(50.0)	9(21.4)	12(28.6)	0(0)	26.1(9.6)	0.028^b^
	Senior high school	225(52.9)	120(28.2)	61(14.4)	19(4.5)	24.3(10.3)	
	Junior College/Technical College	111(40.2)	100(36.2)	51(18.5)	14(5.1)	26.6(9.3)	
	Vocational School/University/Graduate School	624(49.6)	328(26.1)	222(17.7)	83(6.6)	25.5(10.4)	
Parent’s marital status	Unmarried	9(47.4)	3(15.8)	5(26.3)	2(10.5)	27.8(12.1)	0.307^a^
	Married	972(49.1)	554(28.0)	341(17.2)	114(5.8)	25.4(10.2)	
Parent’s sports level	no competition experience	376(49.3)	217(28.4)	123(16.1)	47(6.2)	25.3(10.2)	0.002^b^
	experience in municipal-level competitions	296(48.0)	165(26.7)	126(20.4)	30(4.9)	25.5(10.2)	
	experience in prefectural-level competitions	247(53.0)	133(28.5)	70(15.0)	16(3.4)	24.6(9.4)	
	experience in national competitions	62(40.3)	42(27.3)	27(17.5)	23(14.9)	28.2(11.8)	
Number of children	1	444(47.2)	268(28.5)	169(18.0)	60(6.4)	25.8(10.3)	0.235^b^
	2	415(50.3)	220(26.7)	144(17.5)	46(5.6)	25.3(10.1)	
	3	105(51.2)	61(29.8)	30(14.6)	9(4.4)	24.6(10.1)	
	over 4	17(58.6)	8(27.6)	3(10.3)	1(3.4)	22.9(10.0)	
Children’s sex	Male	616(48.6)	362(28.6)	212(16.7)	77(6.1)	25.5(10.2)	0.665^a^
	Female	365(49.8)	195(26.6)	134(18.3)	39(5.3)	25.3(10.3)	
Children’s sports level	no competition experience	310(50.1)	175(28.3)	101(16.3)	33(5.3)	24.9(10.4)	0.104^b^
	experience in municipal-level competitions	421(48.4)	251(28.9)	152(17.5)	46(5.3)	25.6(9.9)	
	experience in prefectural-level competitions	200(49.1)	113(27.8)	71(17.4)	23(5.7)	25.4(10.3)	
	experience in national competitions	50(48.1)	18(17.3)	22(21.2)	14(13.5)	27.5(11.3)	

* (a) t-test, (b) ANOVA.

Table 3 shows the results of the logistic regression analysis on the relationship between parents’ health literacy and basic information and whether they want their children to receive medicines, vaccines, supplements, and energy drinks. When parents’ health literacy was high, their children’s willingness to receive prescription oral medicines (OR = 1.025, 95% confidence interval [95% CI]: 1.016–1.035), non-prescription oral medicines (OR = 1.012, 95% CI: 1.003–1.021), prescription herbal medicines (OR = 1.021, 95% CI: 1.021–1.030), non-prescription herbal medicines (OR = 1.012, 95% CI: 1.003–1.021), and vaccines (OR = 1.025, 95% CI: 1.016–1.035) increased. Conversely, their children’s willingness to receive energy drinks (OR = 0.990, 95% CI: 0.980–1.000) significantly decreased. Among parents whose children had high levels of athleticism, the willingness to receive only oral prescription medicines for their children decreased (OR = 0.886, 95% CI: 0.791–0.992) and that to receive supplements (OR = 1.492, 95% CI: 1.330–1.673) and energy drinks (OR = 1.480, 95% CI: 1.307–1.676) significantly increased.

**Table 3:** Logistic regression analysis of the associations between related factors and the parents’ intentions for children to receive medicines, vaccines, supplements, and energy drinks

Dependent variables	Independent variables	OR	95% CI	p-value
Oral prescription medicine				
	Parent’s health literacy	1.025	1.016–1.035	< 0.001
	Parent’s sex	1.598	1.315–1.941	< 0.001
	Children’s sports level	0.886	0.791–0.992	0.036
Oral over-the-counter medicine				
	Parent’s health literacy	1.012	1.003–1.021	0.008
	Parent’s age	0.982	0.970–0.994	0.004
Herbal prescription medicine				
	Parent’s health literacy	1.021	1.012–1.030	< 0.001
	Parent’s sex	1.306	1.082–1.577	0.005
	Parent’s age	0.986	0.973–0.999	0.039
Herbal over-the-counter medicine				
	Parent’s health literacy	1.012	1.003–1.021	0.007
	Parent’s sex	0.755	0.630–0.905	0.002
	Parent’s age	0.984	0.971–0.996	0.009
Vaccine				
	Parent’s health literacy	1.025	1.016–1.035	< 0.001
	Parent’s sex	1.215	1.005–1.468	0.045
	Children’s age	0.969	0.941–0.997	0.031
Supplement				
	Parent’s age	0.979	0.965–0.992	0.002
	Children’s sports level	1.492	1.330–1.673	< 0.001
Energy drink				
	Parent’s health literacy	0.990	0.980–1.000	0.041
	Parent’s age	0.969	0.952–0.986	0.001
	Parent’s marital status	0.303	0.120–0.768	0.012
	Children’s age	1.057	1.015–1.101	0.007
	Children’s sports level	1.480	1.307–1.676	< 0.001

*Binomial logistic regression analysis (stepwise method)

** OR: Odds Ratio, CI: Confidence Interval

*** Dummy variable for dependent variable: Parents’ intention to receive oral medicines (both prescription and over-the-counter), herbal medicines (both prescription and over-the-counter), vaccines, supplements, and energy drinks for children (1: not wanting to receive, 2. want to receive)

**** Dummy variable for independent variable: Parents’ sex (1: male, 2: female), marital status (1: unmarried, 2: married), children’s sex (1: male, 2: female), and children’s sports level (1: no competition experience, 2: experience in municipal-level competitions, 3: experience in prefectural-level competitions, and 4: experience in national competitions)

## Discussion

This cross-sectional study focused on determining factors that influence parental preferences regarding the intake of foods and medications related to the health of adolescent athletes. It aimed to clarify whether the health literacy of parents of adolescent athletes was related to their children’s willingness to receive medicines, vaccines, supplements, and energy drinks. This study found that parental health literacy was related to their willingness to receive medicines, vaccines, and energy drinks for their children.

### Health literacy of parents and willingness to have their children receive medicines, supplements, and vaccines

The average health literacy score of the respondents in this study was almost consistent with the results of previous studies conducted among non-athlete adults in Japan [16]. Similarly, the average health literacy score of females was significantly higher than that of males [16]. Sex-related disparities in health literacy differ across countries; however, no sex-associated disparities in digital health literacy were reported in a 2023 meta-analysis [20].

Parents with high health literacy were significantly more likely to have their children receive all medicines, including herbal medicines and vaccines. It has been pointed out that health literacy is related to the proper use of medicines, and not only in patients with chronic diseases, but also in the general population with high health literacy use medicines appropriately [21–23]. In addition, previous research has shown that parental drug literacy is related to the choice of over-the-counter drugs administered to their pre-school children and their knowledge of proper drug use [24].

Health literacy has also been reported to be associated with children’s vaccination intentions, and parents with low health literacy hesitate to get their children vaccinated [25, 26]. Health literacy helps people identify correct information and make appropriate health-related decisions [27]. Therefore, people with high health literacy may better understand the need for medicines and vaccines and make decisions more easily.

However, parents’ high health literacy resulted in a reluctance to receive nutritional drinks for their children (OR = 0.990, 95%CI: 0.980–1.000). An Australian study found that consumption of sweetened energy drinks among secondary school adolescents decreased as their nutritional education increased [28]. From this, it is expected that the improvement of health literacy through nutrition education will curb the excessive consumption of energy drinks, which has become a problem in the recent years [7, 29].

## Children’s athletic levels and parents’ intentions to receive medicines and supplements for children

Parents expressed negative feelings regarding their children’s prescription medicine intake as their children’s athletic performance increased (OR = 0.886, 95%CI: 0.791–0.992). This may have been because they were aware of the doping tests. However, it is necessary to enhance the follow-up by medical staff to avoid limiting the medicine intake necessary for children. In contrast, parents were positive about their children’s intake of supplements (OR = 1.492, 95%CI: 1.330–1.673) and energy drinks (OR = 1.480, 95%CI: 1.307–1.676) when their children’s athletic levels were high. Previous studies revealed that elite athletes with high levels of competition consume many supplements [6, 30]. A survey of long-distance runners in Spain revealed that 70% of athletes take supplements for performance improvement, whereas 80% of the general population take supplements for health maintenance [31, 32]. Considering that both elite athletes and their parents are positive about the consumption of supplements, parents and children may share a positive attitude toward supplements. However, supplements may contain prohibited substances, which can lead to violations of the anti-doping rule [3, 4]. Therefore, it is necessary to raise parents’ awareness regarding the risk of unintended doping from supplements.

The sports pharmacist system in Japan promotes anti-doping activities and is responsible for children’s health education in schools [33, 34]. Such activities can help disseminate knowledge about the intake of medicines, vaccines, supplements, and energy drinks to young athletes and their parents.

### Limitations of this study

One of the weaknesses of this study is that it was a cross-sectional study; therefore, causal relationships could not be determined. To overcome this limitation, future research should employ a longitudinal design. Second, because we were unable to investigate the respondents’ socioeconomic status other than educational background and marital status, we could not determine the impact of socioeconomic status, such as income and living conditions. However, the trends in health literacy observed in this study are similar to those of previous studies that considered the level of income the present study had no significant bias [16]. In addition, because we were unable to investigate the medicines, vaccines, and supplements consumed by the children, we do not know the relationship between the intention of parents and the intake of medicines and supplements by children. Further research should investigate the interplay between parental factors and children’s actual intake of medications, supplements, energy drink, and vaccines. Lastly, despite recruiting respondents equally across sex and age, this survey was conducted on a first-come, first-served basis by a panel of online survey companies. Therefore, only those who were willing to participate were included, indicating a potential bias, compared to the case for surveys with randomly selected respondents.

## Conclusions

High-level parental health literacy among adolescent athletes was associated with their children having positive willingness to receive medications and vaccines, but with negative willingness to receive energy drinks. Parents whose children had a high level of athleticism were hesitant about receiving prescription medicines for their children but had positive intentions about taking supplements and energy drinks.

It is important to promote health education because health literacy helps people make appropriate choices regarding correct information and health-related decisions. Healthcare providers should provide adequate counseling to parents of adolescent athletes to reassure their children about the medications they require. In addition, based on the cases of anti-doping rule violations following consumption of supplements, it is necessary to raise awareness of the dangers of unintended doping among parents. In future, prospective study to examine for causal relationship between parental factors and children’s actual intake of medication, supplements, energy drink, and vaccines should be implemented.

### Electronic supplementary material

Below is the link to the electronic supplementary material.

Supplementary Material 1

## Data Availability

Please contact the corresponding author for inquiries regarding the survey. Due to the privacy of the data, the research team could not share the data publicly.

## Abbreviations

OR: Odds ratio
CI: Confidence interval
SD: Standard deviation
HLS-EU: European Health Literacy Survey Questionnaire
