# Supplementary material for: Is health literacy of adolescent athletes’ parents whose children belonged to sports clubs related to their children’s intention to receive medications, vaccines, supplements, and energy drinks? A cross-sectional study
Source: BMC Public Health. 2024 Jan 22;24:257. doi: 10.1186/s12889-024-17746-0 (PMC10804555; doi:10.1186/s12889-024-17746-0)
Supplement: Supplementary file 1 — Supplementary Material 1 [file 12889_2024_17746_MOESM1_ESM.docx]

HLS-EU-Q16 Japanese version

|  | | とても簡単  Very easy | やや 簡単  Fairly easy | やや 難しい  Fairly difficult | とても難しい  Very difficult | わからない/あてはまらない  Don’t know/Not applicable |
| --- | --- | --- | --- | --- | --- | --- |
| 1 | 気になる病気の治療に関する情報を見つけるのは  Finding information on treatments of illnesses that concern you | 1 | 2 | 3 | 4 | 5 |
| 2 | 病気になった時、専門家（医師、薬剤師、心理士など）に相談できるところを見つけるのは  Finding out where to get professional help when you are ill | 1 | 2 | 3 | 4 | 5 |
| 3 | 医師から言われたことを理解するのは  Understanding what your doctor says to you | 1 | 2 | 3 | 4 | 5 |
| 4 | 処方された薬の服用方法について、医師や薬剤師の指示を理解するのは  Understanding your doctor’s or pharmacist’s instructions on how to take a prescribed medicine | 1 | 2 | 3 | 4 | 5 |
| 5 | 別の医師からセカンド・オピニオン(主治医以外の医師の意見)を得る必要があるかどうかを判断するのは  Judging when you may need to get a second opinion from another doctor | 1 | 2 | 3 | 4 | 5 |
| 6 | 自分の病気に関する意思決定をする際に、医師から得た情報を用いるのは  Using information the doctor gives you to make decisions about your illness | 1 | 2 | 3 | 4 | 5 |
| 7 | 医師や薬剤師の指示に従うのは  Using information the doctor gives you to make decisions about your illness | 1 | 2 | 3 | 4 | 5 |
| 8 | ストレスや抑うつなどの心の健康問題への対処方法に関する情報を見つけるのは  Finding information on how to manage mental health problems such as stress or depression | 1 | 2 | 3 | 4 | 5 |
| 9 | 喫煙、運動不足、お酒の飲み過ぎなどの生活習慣が健康に悪いと理解するのは  Understanding health warnings about behavior such as smoking, low physical activity and excessive drinking | 1 | 2 | 3 | 4 | 5 |
| 10 | 検診（乳房検査、血糖検査、血圧）が必要な理由を理解するのは  Understanding why you need health screenings | 1 | 2 | 3 | 4 | 5 |
| 11 | メディアから得た健康リスクの情報が信頼できるかどうかを判断するのは  Judging if the information on health risks in the media is reliable | 1 | 2 | 3 | 4 | 5 |
| 12 | メディア（新聞、ちらし、インターネット、その他のメディア）から得た情報をもとに、病気から身を守る方法を決めるのは  Deciding how you can protect yourself from illness based on information in the media | 1 | 2 | 3 | 4 | 5 |
| 13 | 心を豊かにする活動について知るのは  Finding out about activities that are good for your mental well-being | 1 | 2 | 3 | 4 | 5 |
| 14 | 健康に関する家族や友人のアドバイスを理解するのは  Understanding advice on health from family members or friends | 1 | 2 | 3 | 4 | 5 |
| 15 | 健康になるためのメディア情報を理解するのは  Understanding information in the media on how to get healthier | 1 | 2 | 3 | 4 | 5 |
| 16 | どの生活習慣（飲酒、食生活、運動など）が自分の健康に関係しているかを判断するのは  Judging which everyday behavior is related to your health | 1 | 2 | 3 | 4 | 5 |
